# The rectification of heterotypic Cx46/Cx50 gap junction channels depends on intracellular magnesium

**DOI:** 10.52601/bpr.2024.240015

**Published:** 2024-10-31

**Authors:** Honghong Chen, Donglin Bai

**Affiliations:** 1 Department of Physiology and Pharmacology, University of Western Ontario, London, Ontario, Canada

**Keywords:** Gap junction channel, Heterotypic gap junction, Rectification, Single channel analysis, Patch clamp

## Abstract

Gap junction (GJ) intercellular communication is crucial in many physiological and pathological processes. A GJ channel is formed by head-to-head docking of two hexameric hemichannels from two neighboring cells. Heterotypic GJ channels formed by two different homomeric connexin hemichannels often display rectification properties in the current–voltage relationship while the underlying mechanisms are not fully clear. Here we studied heterotypic Cx46/Cx50 GJs at a single GJ channel level. Our data showed unitary Cx46/Cx50 GJ channel conductance (γ_j_) rectification when 5 mmol/L Mg^2+^ was included in the patch pipette solution, while no γ_j_ rectification was observed when no Mg^2+^ was added. Including 5 mmol/L Mg^2+^ in pipette solution significantly decreased the γ_j_ of homotypic Cx46 GJ with little change in homotypic Cx50 γ_j_. A missense point variant in Cx46 (E43F) reduced the Mg^2+^-dependent reduction in γ_j_ of Cx46 E43F GJ, indicating that E43 might be partially responsible for Mg^2+^-dependent decrease in γ_j_ of Cx46. A comprehensive understanding of Mg^2+^ modulation of GJ at the individual channel level is useful in understanding factors in modulating GJ-mediated intercellular communication in health and diseases.

## INTRODUCTION

Gap junctions (GJs) are clusters of intercellular channels that provide direct passage of ions, nutrients, metabolic wastes, and small signaling molecules between adjacent cells to regulate tissue/organ homeostasis (Goodenough and Paul 2009). A GJ channel is formed from two head-to-head docked connexons (known as hemichannels) and each connexon is composed of six connexins (Goodenough and Paul 2009). Twenty-one human genes and twenty mouse genes are identified to encode for connexins and all of the connexins share the same topological structures with four transmembrane domains (M1–M4), two extracellular loops (E1 and E2), one cytoplasmic loop (CL), and amino terminus (NT) and carboxyl terminus (CT) in the cytosol (Kumar and Gilula 1996). Connexin expression is tissue cell-specific, and each tissue cell often expresses one or more connexins, leading to the formation of homomeric homotypic, homomeric heterotypic, and/or heteromeric heterotypic GJs (Saez *et al.* 2003; Goodenough and Paul 2009). Depending on the component connexins, different GJs showed a variety of properties and can be modulated by many factors, including intracellular pH, transjunctional voltage (*V*_j_), post-translational modifications, and intracellular divalent cations, such as Ca^2+^ and Mg^2+^ (Peracchia *et al.* 2000; Bukauskas and Verselis 2004; Lampe and Lau 2004; Bargiello and Brink 2009).

Early studies documented that elevation of intracellular Ca^2+^ concentrations (*i*.*e*., [Ca^2+^]_i_) leads to GJ uncoupling in various cells (Loewenstein *et al.* 1967; Oliveira-Castro and Loewenstein 1971; Noma and Tsuboi 1987). Similar to this action of [Ca^2+^]_i_, [Mg^2+^]_i_ can also uncouple GJs at millimolar concentrations, which are much higher than those required for [Ca^2+^]_i_ to reduce GJ coupling (Oliveira-Castro and Loewenstein 1971; Peracchia and Peracchia 1980; Noma and Tsuboi 1987; Matsuda *et al.* 2010). More recent studies on recombinantly expressed connexins showed that the coupling conductance of the GJs formed by Cx26, Cx32, Cx36, Cx43, Cx45, or Cx47 were all reduced in 5 mmol/L [Mg^2+^]_i_ from the initial coupling level (Palacios-Prado *et al.* 2013). However, a much lower [Mg^2+^]_i_ (0.1–0.01 mmol/L) was found to enhance Cx36 GJ coupling conductance, but not other tested GJs (Palacios-Prado *et al.* 2013). This unique [Mg^2+^]_i_ modulation on Cx36 was mapped to the residue Asp47 (D47) of Cx36, which is at the border of M1 and E1 domains (Palacios-Prado *et al.* 2014). Interestingly, the border of M1 and E1 domains has been shown to be the site for Ca^2+^ binding in Cx26 GJs (E42, G45 and E47 of Cx26), and one of these residues (G45) is the equivalent residue of D47 of Cx36 (Bennett *et al.* 2016). Additionally, a Ca^2+^ binding site responsible for hemichannel gating was identified at the residues (E47 and D50) in Cx26 (Lopez *et al.* 2016) and also at the M1–E1 border region. The M1–E1 region in other connexins, including Cx46 and Cx50, are highly conserved and a cluster of three or more negatively charged residues were found to be the pore lining/accessible residues in the high-resolution structure models (Myers *et al.* 2018; Flores *et al.* 2020). Pore lumen accessibility and multiple negative charges in Cx46 and Cx50 GJs at this M1–E1 border region suggest that this domain may also be able to bind divalent cations, including Mg^2+^. Functional study with different concentrations of [Mg^2+^]_i_ showed a dose-dependent reduction of the unitary channel conductance (*γ*_j_) of Cx50 GJ (Tejada *et al.* 2018). However, it is not clear if [Mg^2+^]_i_ can modulate the *γ*_j_ of homotypic Cx46 or heterotypic Cx46/Cx50 GJs in a similar way.

The human eye lens is an avascular organ, and its development and transparency are dependent on microcirculations mediated by GJ networks (Mathias *et al.* 2010). Cx46, Cx50 and Cx43 are expressed in the lens with both Cx50 and Cx43 in the epithelial cells and the Cx46 and Cx50 in the lens fiber cells which make up the bulk of the lens (Beyer *et al.* 1989; Paul *et al.* 1991; White *et al.* 1992; Mathias *et al.* 2010). Over one hundred mutations in the genes encoding either Cx46 or Cx50 have been linked to congenital cataracts (Beyer *et al.* 2013; Bai *et al.* 2021). Many of these mutations are co-segregated with the disease for multiple generations (Beyer *et al.* 2013; Ceroni *et al.* 2019). Functional studies of these cataracts linked Cx46 or Cx50 mutants revealed a loss of GJ function as a common mechanism for these mutants (Pal *et al.* 1999; Tong *et al.* 2013; Schadzek *et al.* 2016; Abrams *et al.* 2018; Li *et al.* 2023). Consistent with these findings, the mouse models with either Cx46 or Cx50 genes knockout also developed cataracts (Gong *et al.* 1997; White *et al.* 1998) and mutations in mouse Cx50 gene also linked to inherited cataracts (Xu and Ebihara 1999; Berthoud *et al.* 2019). In summary, compelling evidence from genetic, functional, and animal models is accumulated for an important role of GJs formed by Cx46 and Cx50 for lens transparency.

Co-expression of Cx46 and Cx50 in the lens fiber cells opens the theoretical possibilities of forming different types of GJs (Mathias *et al.* 2010). Studies in native lens tissues revealed that heteromeric GJs with both Cx46 and Cx50 were formed (Konig and Zampighi 1995; Jiang and Goodenough 1996; Myers *et al.* 2018). Recombinant expression studies indicate that functional homomeric heterotypic Cx46/Cx50 GJs can be formed in model cells (White *et al.* 1994; Hopperstad *et al.* 2000; Wong *et al.* 2024). Here we studied homomeric heterotypic Cx46/Cx50 GJs at a single channel level to determine the *γ*_j_s under Mg^2+^-free and added Mg^2+^ intracellular solutions. To align our functional study with the high resolution of sheep Cx46 and Cx50 GJ structure models (Myers *et al.* 2018; Flores *et al.* 2020), we used the sheep version of Cx46 and Cx50 to evaluate the effect of [Mg^2+^]_i_ on the *γ*_j_ of these GJs. Our results showed that the rectification property of heterotypic Cx46/Cx50 GJs depended on the presence of intracellular Mg^2+^. This is likely due to a higher sensitivity of Cx46 GJ to [Mg^2+^]_i_. Studying the mechanisms of [Mg^2+^]_i_ modulation in these lens GJs may shed light on the divalent cation modulation of many GJs in health and disease conditions.

## RESULTS

### Cx46/Cx50 GJ showed rectification in the presence of Mg^2+^

**Figure 1:**
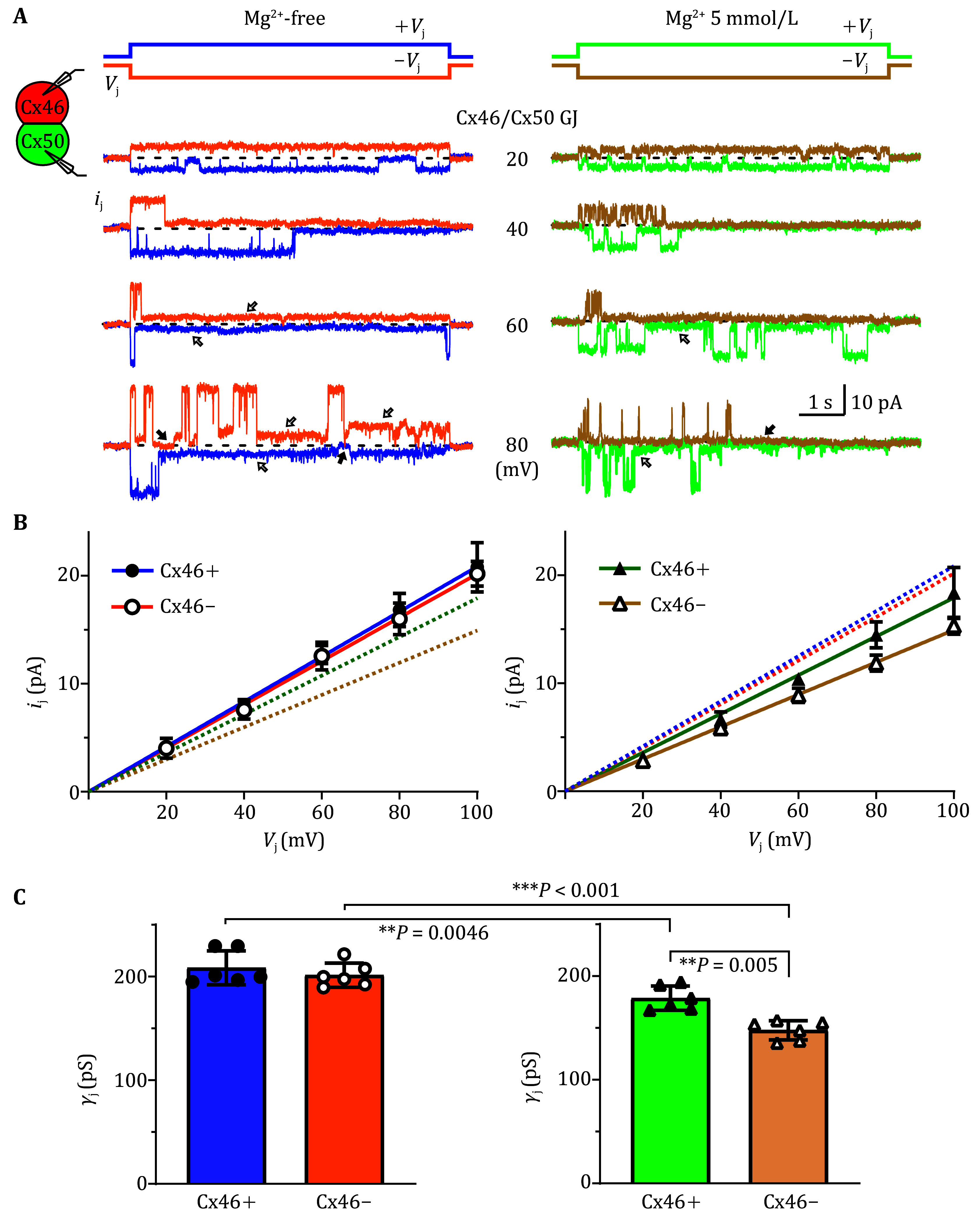
Single channel properties of heterotypic Cx46/Cx50 GJ in the absence and presence of intracellular Mg^2+^. **A** Dual whole cell patch clamp technique was used to measure single gap junction channel current (*i*_j_) in response to the indicated *V*_j_ in heterotypic N2A cell pairs expressing sheep Cx46 in one (with untagged reporter DsRed) and sheep Cx50 in the other of the cell pair (with untagged reporter GFP). In the absence of intracellular Mg^2+^ (Mg^2+^-free), the absolute *i*_j_s at the main conducting state were increased proportionally to the absolute *V*_j_s at both +*V*_j_ (purple color) and –*V*_j_ (orange color) polarities. Transitions from the main conducting state to the residue conducting state (open arrows) or fully closed state (filled arrow) could be observed especially on the *i*_j_s induced by ±40 or higher *V*_j_s. The *i*_j_s in the presence of 5 mmol/L Mg^2+^ were slightly lower in the amplitude for both +*V*_j_ (in green color) and –*V*_j_s (in brown color). **B** Main conducting state *i*_j_s were plotted against the *V*_j_ for each *V*_j_ polarity in the absence and presence of Mg^2+^. Linear regressions of the *i*_j_–*V*_j_ plots were used to obtain slope single channel conductance (*γ*_j_) and the regression lines showed in different colors in the absence of Mg^2+^ (Cx46+ shown in purple and Cx46– shown in orange color) and the presence of 5 mmol/L Mg^2+^ (Cx46+ shown in green and Cx46– shown in brown color). The dotted lines were included to highlight the differences in slope *γ*_j_s. **C** Bar graphs illustrate the average *γ*_j_ for heterotypic Cx46/Cx50 GJ at different *V*_j_ polarities as indicated. The *γ*_j_s in the presence of 5 mmol/L Mg^2+^ displayed a statistical difference between Cx46+ and Cx46– (**P < 0.01). The *γ*_j_s of Cx46+ and Cx46– in the presence of 5 mmol/L Mg^2+^ were lower than their corresponding *γ*_j_s in the absence of Mg^2+^

To study homomeric heterotypic Cx46/Cx50 GJ channels, we mixed cells expressing Cx46-IRES-DsRed with cells expressing Cx50-IRES-GFP and selected cell pairs with successfully expressing the fluorescent reporters, one red (DsRed+) and the other one green (GFP+) for dual whole-cell voltage clamp experiment. Transjunctional voltage (*V*_j_) was generated by a series of voltage pulses delivered in one cell of the pair while holding the other cell of the pair constantly at zero to record transjunctional current. Cell pairs with only 1–2 functional GJ channels were selected for further analysis. Representative unitary transjunctional currents (*i*_j_s) in response to a series of *V*_j_s are shown in Fig. 1 under Mg^2+^-free intracellular solution (ICS). The *i*_j_s of the main conducting state were plotted with *V*_j_s and a linear regression of *i*_j_–*V*_j_ plot was used to obtain slope single channel conductance (*γ*_j_) for two current flow directions. The *γ*_j_ of Cx46+ (defined as relative +*V*_j_s on the Cx46-expressing cell comparing to the Cx50-expressing cell) was 208.4 ± 6.7 pS (*n* = 6), which is not significantly different from the *γ*_j_ (201.4 ± 4.8 pS, *n* = 6, *P* = 0.50) of Cx46– (relative –*V*_j_s on the Cx46 cell comparing to the Cx50 cell) in Mg^2+^-free ICS. This is surprising considering a previous study reported rectification of heterotypic rodent Cx46/Cx50 GJ (Hopperstad *et al.* 2000). With a closer comparison of the experimental conditions, we found that the ICS in that study contained 3 mmol/L Mg^2+^, which could modify the *γ*_j_ as reported in Cx50 GJ (Tejada *et al.* 2018). To test this possibility, we used ICS containing 5 mmol/L Mg^2+^ in the form of MgATP to repeat the previous experiment. With 5 mmol/L Mg^2+^ in ICS, we observed rectification for heterotypic Cx46/Cx50 GJ with the *γ*_j_ of Cx46+ (178.7 ± 4.8 pS, *n* = 6) which was significantly higher than the *γ*_j_ of Cx46– (147.8 ± 3.8 pS, *n* = 6, **P = 0.005, Fig. 1). Both Cx46+ and Cx46– *γ*_j_s in 5 mmol/L Mg^2+^ were lower than the corresponding *γ*_j_s in the absence of Mg^2+^ as indicated in Fig. 1C.

### The *γ*_j_s of homotypic Cx46 GJs were decreased in the presence of intracellular Mg^2+^

**Figure 2:**
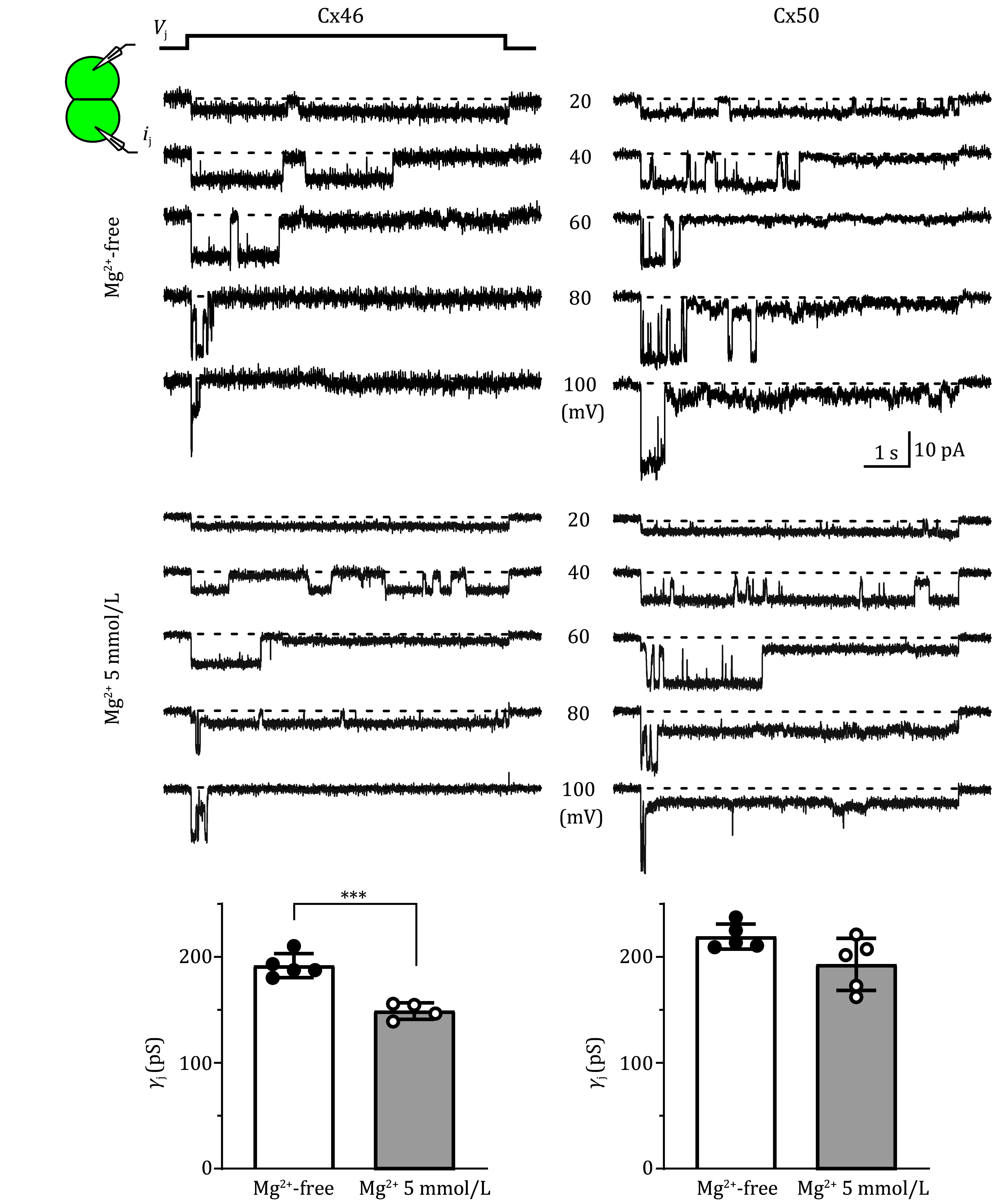
Homotypic Cx46 and Cx50 GJs showed different sensitivity to intracellular Mg^2+^. Unitary transjunctional currents (*i*_j_s) recorded from cell pairs expressing Cx46 (left panels) or Cx50 (right panels) in the absence (Mg^2+^ free, black traces) or presence (5 mmol/L Mg^2+^, grey traces) of intracellular Mg^2+^ to different *V*_j_ pulses. The average slope *γ*_j_s of Cx46 and Cx50 GJs were plotted as bar graphs in the absence (open bars) and presence (grey bars) of 5 mmol/L Mg^2+^ as indicated. Statistical significance is indicated (***P < 0.001)

It is interesting to observe a [Mg^2+^]_i_-dependent rectification of heterotypic Cx46/Cx50 GJ channels. To explore the underlying mechanisms, we studied the *γ*_j_s of homotypic Cx46 and Cx50 GJs in the absence and presence of Mg^2+^. As shown in Fig. 2, *i*_j_s of homotypic Cx46 and Cx50 GJs were recorded with Mg^2+^-free and Mg^2+^-containing (5 mmol/L) ICS. The slope *γ*_j_s were obtained by linear regression of *i*_j_–*V*_j_ plot for each cell pair of these homotypic GJs and then plotted as a bar graph (Fig. 2 bottom panels). The *γ*_j_ of Cx46 GJ was 192 ± 5.1 pS (*n* = 5) in the absence of Mg^2+^, which was significantly higher than the *γ*_j_ in the presence of 5 mmol/L Mg^2+^ (149 ± 3.9 pS, *n* = 4, ***P < 0.001, Fig. 2). In other words, 5 mmol/L Mg^2+^ caused an average 22% reduction of the slope *γ*_j_ of Cx46 GJ. Different from Cx46 GJ, the *γ*_j_ of Cx50 GJ was 219 ± 5.3 pS (*n* = 5) in the absence of Mg^2+^, which was not statistically different from the *γ*_j_ in the presence of 5 mmol/L Mg^2+^ (193 ± 11 pS, *n* = 5, *P* = 0.065, Fig. 2). Though an apparent decrease in the slope *γ*_j_ of Cx50 GJ in 5 mmol/L Mg^2+^ was observed (12%).

### The 43^rd^ residue in Cx46 GJ could be partially responsible for its Mg^2+^ modulation

**Figure 3:**
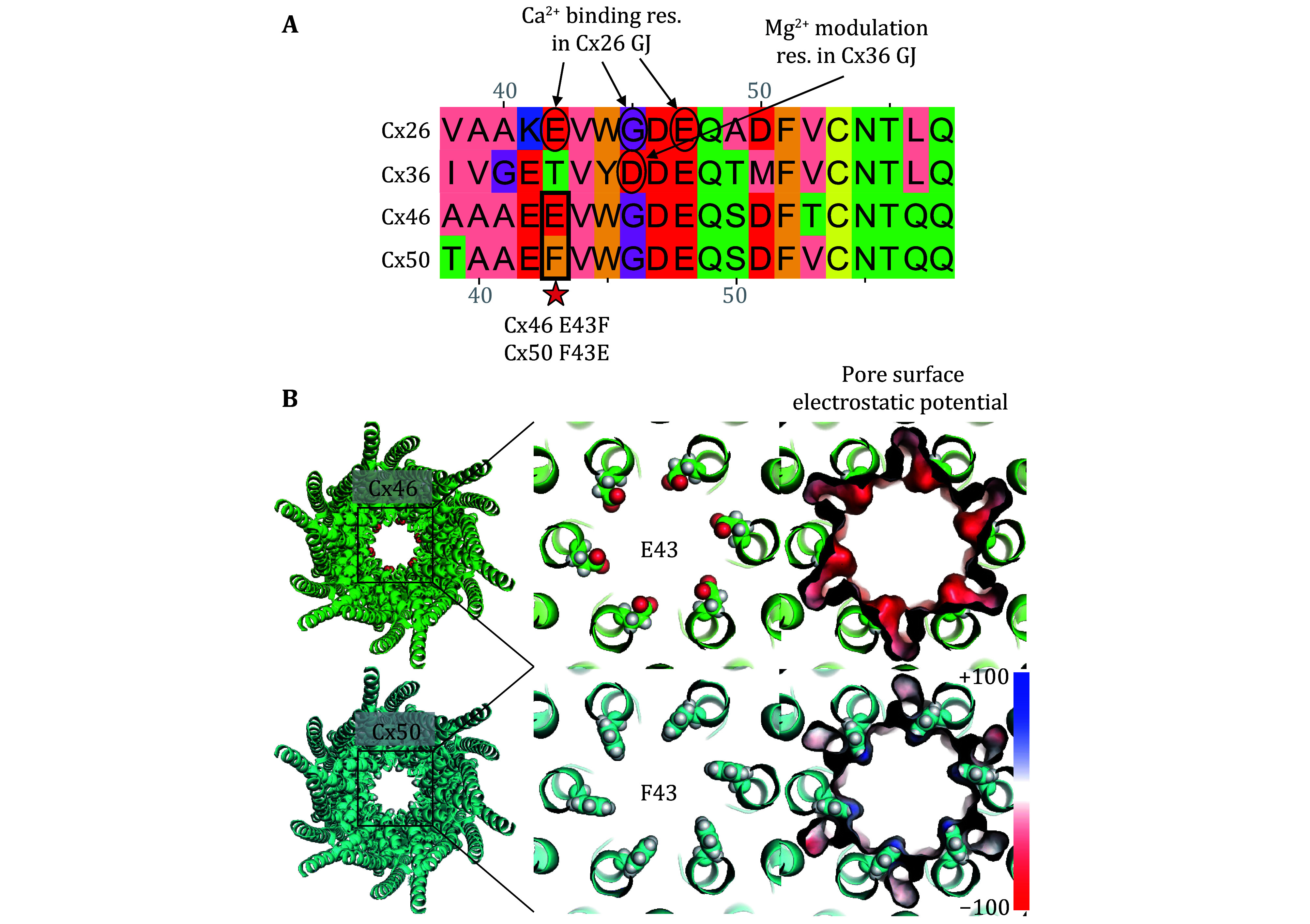
Sequence alignment and possible residues involved in divalent cation binding in selected connexins. **A** Protein sequence alignment of human Cx26, Cx36, and sheep Cx46 and Cx50 near the border between the first transmembrane (M1) domain and the first extracellular (E1) domain. Ca^2+^-binding residues in Cx26 and the key residue for Mg^2+^-dependent modulation in Cx36 are indicated by circles and arrows. The residue numbering on the top follows that of Cx26 and the residue numbering at the bottom follows those of Cx46 and Cx50. Residues at the 43^rd^ position of Cx46 and Cx50 are different (indicated by a rectangle box) and our designed variants are indicated below the alignment. **B** Structure models of Cx46 and Cx50 GJs (left panels), based on 7JKC and 7JJP (Flores *et al.* 2020), respectively, to show the sidechain of Cx46 E43 and Cx50 F43 are lining the pore (zoomed in view middle panels). The Cx46 E43 vacuum pore surface electrostatic potential showed stronger negative potential (red) than those of Cx50 F43 (mostly white with slightly blue, right panels)

To explore the underlying structural mechanism of Mg^2+^ modulation of Cx46 *γ*_j_, we looked for sequence differences between Cx46 and Cx50 at a region identified to be important for divalent cation binding/modulation in other GJs, specifically Mg^2+^ modulation site in Cx36 and Ca^2+^-binding site in Cx26 GJs (Palacios-Prado *et al.* 2014; Bennett *et al.* 2016). We first aligned these connexins at the region of E1–M1 domain region (Fig. 3A). The previously identified Ca^2+^-binding residues (E42, G45, and E47) in Cx26 GJ overlaps with the key residue identified for Mg^2+^-modulation in Cx36 (D47 which is aligned with G45 of Cx26) (Palacios-Prado *et al.* 2014; Bennett *et al.* 2016). Both Cx46 and Cx50 sequences in this region are highly homologous and aligned very well with either Cx26 or Cx36 (Fig. 3A). The only obvious difference was observed on the 43^rd^ position (the equivalent residue in Cx26, E42, involved in binding Ca^2+^) where in Cx46 was also a negatively charged glutamate (E43) whereas in Cx50 was a non-charged aromatic phenylalanine (F43). According to the high-resolution structures of Cx46 and Cx50 GJs (Myers *et al.* 2018; Flores *et al.* 2020), the 43^rd^ residue in Cx46 and Cx50 lines the permeation passage. A ring of six negatively charged E43 in each of the Cx46 connexons showed much more negative pore surface electrostatic potential attracting divalent cations such as Mg^2+^ (Fig. 3B), whereas in Cx50 connexon a ring of six non-charged F43 produced a neutral pore surface electrostatic potential unlikely to attract Mg^2+^ at this position (Fig. 3B). Based on these structural insights and the equivalent residue involvement in Ca^2+^-binding in Cx26 GJ, we hypothesize that Cx46 E43 is one of several residues involved in Mg^2+^-binding in Cx46 GJ and a missense mutation on this residue, E43F in Cx46, could reduce the Mg^2+^ modulation at this site. Reversely, a missense mutant in Cx50, F43E, could increase the pore surface negative electrostatic potential to attract Mg^2+^ to enhance Mg^2+^ modulation.

**Figure 4:**
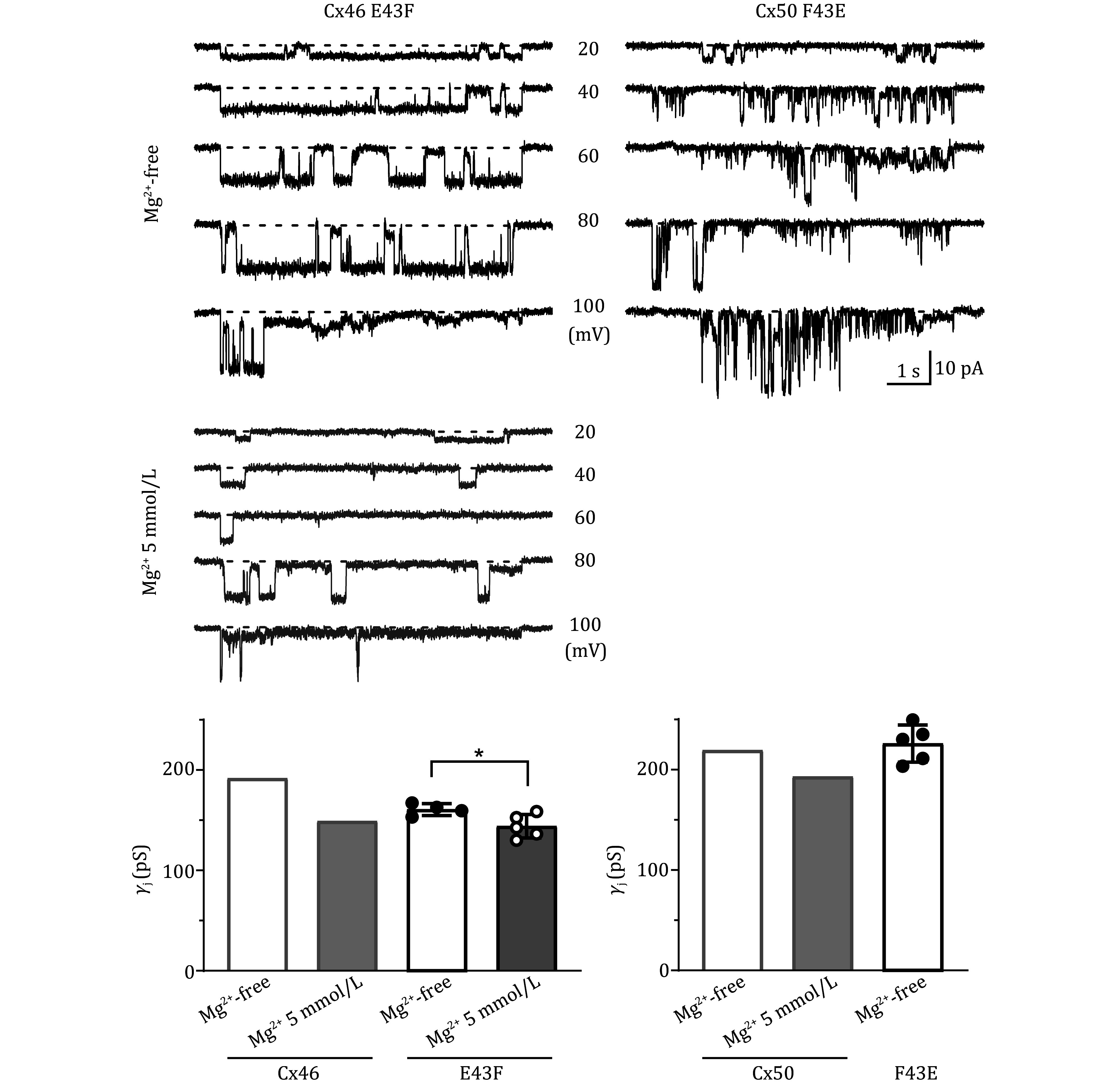
Cx46 E43F showed an apparently lower percentage block by [Mg^2+^]_i_ compared to wildtype Cx46. Representative unitary transjunctional currents (*i*_j_s) recorded from cell pairs expressing Cx46 E43F (left top panel) or Cx50 F43E (right top panel) in the absence (Mg^2+^ free, black traces) or presence (5 mmol/L Mg^2+^, middle panel with grey traces) of intracellular Mg^2+^ to different *V*_j_ pulses. In the presence of 5 mmol/L Mg^2+^ in the ICS, the Cx50 F43E failed to form any function GJs. The average slope *γ*_j_s of Cx46 E43F and Cx50 E43F GJs were plotted as bar graphs in the absence (open bars) and presence (grey bar) of 5 mmol/L Mg^2+^ as indicated. For easier comparison, the average slope *γ*_j_ of Cx46 and Cx50 GJs in the absence (Mg^2+^-free) and the presence of 5 mmol/L Mg^2+^ are included in these bar graphs (from Fig. 2 with grey outlines). Statistical significance is indicated (*P = 0.036). Data points represent the number of cell pairs

Our experimental results showed that expressing Cx46 E43F formed functional GJs with a slope *γ*_j_ of 160.7 ± 2.9 pS (Fig. 4, *n* = 4) in Mg^2+^-free intracellular solution, which was reduced to 144 ± 5.2 pS (*n* = 5, *P = 0.036) when 5 mmol/L Mg^2+^ was included in the ICS (Fig. 4). Cx50 F43E was also formed functional GJs with a slope *γ*_j_ of 226 ± 8.3 pS (Fig. 4, *n* = 5) in Mg^2+^-free ICS. However, the channel rarely stayed at the fully open state for any extended period (Fig. 4). When recording with ICS containing 5 mmol/L Mg^2+^, no functional GJs could be identified, which could be due to the Cx50 F43E GJ showing an increased sensitivity to Mg^2+^ binding to promote GJ channel closure.

## DISCUSSION

The present study identified an intracellular Mg^2+^ concentration dependent rectification of heterotypic Cx46/Cx50 GJs at individual channel levels. Specifically, the single channel conductance (*γ*_j_) of Cx46/Cx50 GJ was significantly higher when the Cx46 cell was positive (defined as Cx46+) than that when the Cx46 cell was negative (Cx46–) with respect to the Cx50 cell in the intracellular solution containing 5 mmol/L Mg^2+^, but in Mg^2+^-free intracellular solution the γ_j_s of Cx46+ and Cx46– were larger and no difference was observed between them. Elevation of [Mg^2+^]_i_ from zero to 5 mmol/L showed a significant reduction in the *γ*_j_ of homotypic Cx46 GJ with little change in the *γ*_j_ of homotypic Cx50 GJ. The [Mg^2+^]_i_-dependent reduction in Cx46 *γ*_j_ was likely due to the E43 of Cx46, as Cx46 E43F showed a reduced [Mg^2+^]_i_-modulation in *γ*_j_. Unfortunately, we could not study the [Mg^2+^]_i_-modulation on Cx50 F43E as the GJ main open state became very unstable in the Mg^2+^-free intracellular solution and no functional GJs could be formed in the presence of 5 mmol/L Mg^2+^. Our study revealed that [Mg^2+^]_i_ could modulate the *γ*_j_ of Cx46 and Cx50 GJs differently leading to [Mg^2+^]_i_-dependent rectification of heterotypic Cx46/Cx50 GJ. Variation in intracellular Mg^2+^ concentrations under physiological and pathological conditions could regulate the *γ*_j_s of homotypic Cx46, heterotypic Cx46/Cx50, and possibly other GJs to fine tune intercellular communication in the lens and other tissues.

The underlying mechanism of how [Mg^2+^]_i_ could modulate Cx46/Cx50 GJ channel rectification is not clear. A previous study showed that the rectification of a heterotypic Cx26/Cx32 GJ channel was due to their differences in ion selectivity of the component hemichannels (Suchyna *et al.* 1999), *i*.*e*., Cx26 GJ showed a strong cation preference while Cx32 GJ did not. When Cx26 and Cx32 hemichannels connected in series forming a GJ channel, the availability of cations and anions in different parts of the heterotypic GJ pore were different leading to current rectification (Suchyna *et al.* 1999). In the case of Cx46 and Cx50 GJs, the permeation ions showed cation preference for both GJs (Trexler *et al.* 1996; Srinivas *et al.* 1999; Sakai *et al.* 2003; Tong *et al.* 2014) and our measured *γ*_j_ of heterotypic Cx46/Cx50 GJ showed little difference with different *V*_j_ in the absence of Mg^2+^. However, when 5 mmol/L Mg^2+^ is added into the intracellular solution, we believe that one or more Mg^2+^ can bind to the channel pore surface where negatively charged residues are enriched, such as near the border of M1–E1 domains (Fig. 3). As Cx46 is predicted to have one additional negatively charged residue (E43, amplified into six in a hexameric Cx46 hemichannel) than Cx50, it is therefore more likely to interact with Mg^2+^ whenever intracellular Mg^2+^ is available. The interaction between Mg^2+^ and the pore lining residues could cause changes in local electrostatic properties, which in turn could alter the local concentrations of cations and anions to change the channel cation/anion preference on the Cx46 half of the heterotypic Cx46/Cx50 GJ, leading to rectification as observed. Consistent with this model, we did observe a significant reduction in the *γ*_j_ (22%) of wildtype Cx46 GJ in the presence of [Mg^2+^]_i_, while little change in the *γ*_j_ of Cx50 GJ was observed. In addition, Cx46 E43F variant GJ is predicted to have a decreased negative electrostatic potential at the M1–E1 border region and its GJ showed a decreased *γ*_j_ reduction (10%) by [Mg^2+^]_i_. Additional experiments with enlarged cations or anions to directly test the cation/anion preference for homotypic Cx46, its variant E43F, and Cx50 GJs would be required to reveal if these GJs’ cation/anion preferences and *γ*_j_s are different with different sized permeation ions.

Most of the intracellular Mg^2+^ ions are in bound form with ATP or enzymes as a co-factor, the free Mg^2+^ concentration was estimated to be only a small fraction of the total intracellular Mg^2+^ (Romani and Scarpa 2000; Maguire and Cowan 2002). This is also the case for our experiments described above. We included 5 mmol/L MgATP in the patch pipette solution and the estimated free [Mg^2+^]_i_ is ~8 times lower (0.6 mmol/L) according to an online program (Maxchelator) with our patch pipette solution (Patton *et al.* 2004). We believe that this estimation is likely an underestimation of [Mg^2+^]_i_ as ATP is not stable in solutions and is likely partially hydrolyzed into ADP and AMP, both of which showed a lower affinity in binding Mg^2+^ than ATP. This free [Mg^2+^]_i_ is well within the range of estimated free concentrations for Mg^2+^ in cytosols for most tissues (0.2–3.5 mmol/L) (London 1991; Chen *et al.* 2003). It is interesting to note that at this free [Mg^2+^]_i_, we did not observe a statistically significant decrease in the *γ*_j_s of Cx50 GJ as we reported earlier (Tejada *et al.* 2018). We believe that several reasons could be responsible for this apparent difference. (1) We did not include any ATP in the patch pipette solution in our previous study, therefore using 1 or 3 mmol/L MgCl_2_ in the patch pipette solution, the Maxchelator estimated free [Mg^2+^]_i_ are around 0.7 or 2.1 mmol/L, respectively. These free concentrations of Mg^2+^ are actually higher than simply including 5 mmol/L MgATP. (2) Our previous study used mouse Cx50 which showed a sequence identity of 87% from sheep Cx50 (the one we used for the current study). With 13% different residues, the sheep GJ structure could be slightly different leading to some differences in Mg^2+^-dependent modulation on *γ*_j_. (3) We could not rule out other additional differences in handling solutions by different researchers for these patch clamp experiments. In addition to modulating the *γ*_j_, higher free [Mg^2+^]_i_ (in the 1–10 mmol/L range) was previously shown to be able to affect *V*_j_-gating properties in Cx36, Cx43, and Cx47 GJs (Palacios-Prado *et al.* 2013). In contrast to these findings, our previous study on mouse Cx50 failed to observe any *V*_j_-gating property change at 3 mmol/L Mg^2+^ without any added ATP (Tejada *et al.* 2018). It is not clear if the *V*_j_-gating properties of Cx46 GJs could be modulated by higher [Mg^2+^]_i_, this needs to be tested experimentally in future studies.

The heterotypic Cx46/Cx50 GJ channel displayed rectification in the presence of [Mg^2+^]_i_, indicating that these heterotypic GJs could play a role in facilitating directional movement of ions. GJs are large pores able to not only permeate ions but also larger organic substrates up to one kilodalton in molecular weight. The rectification of ionic currents in the heterotypic GJ channels could be amplified to promote directional permeation of larger organic substrates, such as nutrients, metabolites, and signaling molecules. Regulating the directional permeation of these molecules through heterotypic GJ channels could be important for GJ-dependent lens circulation to keep this avascular organ transparent. Currently, it is not clear how Cx46 and Cx50 are assembled into different types of heteromeric and/or heterotypic GJs. Heterotypic Cx46/Cx50 GJ might not be the dominant GJ type in lens fiber cells for directional circulation. Whether complex heteromeric GJs formed by a mixture of Cx46 and Cx50 also display rectification properties will require properly designed experiments to demonstrate.

Elevation of either [Ca^2+^]_i_ or [Mg^2+^]_i_ has been shown to eliminate/reduce GJ function in cells expressing different connexins (Loewenstein *et al.* 1967; Oliveira-Castro and Loewenstein 1971; Noma and Tsuboi 1987; Peracchia *et al.* 2000; Peracchia 2004; Chen *et al.* 2011; Palacios-Prado *et al.* 2013). However, some key differences are noted between [Ca^2+^]_i_ and [Mg^2+^]_i_ modulations on GJs. (1) The concentrations of intracellular Ca^2+^ and Mg^2+^ are quite different in tissue cells under physiological conditions. The endogenous [Ca^2+^]_i_ is in the tens to hundreds of nano molar (nmol/L) range and [Mg^2+^]_i_ is in the sub- or low-millimolar range (Grubbs 2002). So the [Mg^2+^]_i_ is at least 1000 times higher than [Ca^2+^]_i_, to affect the GJ channel function. (2) Elevation of [Ca^2+^]_i_ to micro molars often leads to the elimination of GJ coupling (Peracchia 2004; Chen *et al.* 2011; Zou *et al.* 2014), while the increase in [Mg^2+^]_i_ to milli molars showed a significantly reduced GJ coupling, but not elimination (Palacios-Prado *et al.* 2013). Previous works indicated that 5 mmol/L free [Mg^2+^]_i_ reduce coupling conductance of GJs of Cx26, Cx32, Cx36, Cx43, Cx45, and Cx47 (Palacios-Prado *et al.* 2013; Palacios-Prado *et al.* 2014). It is not clear whether this reduction is due to a decrease in GJ channel open probability, *γ*_j_, or both. Our previous works showed that [Mg^2+^]_i_ (without any added ATP in patch pipette) dose dependently reduced *γ*_j_ of Cx50 (Tejada *et al.* 2018) and now we showed that Cx46 *γ*_j_ was more sensitive to [Mg^2+^]_i_ (together with ATP) than that of Cx50 *γ*_j_ leading to the rectification of heterotypic Cx46/Cx50 γ_j_. (3) The underlying molecular/structural mechanisms of how Ca^2+^ modulates GJs are not fully clear. Depending on the [Ca^2+^]_i_, Ca^2+^ could have an indirect effect on GJ via calmodulin (Peracchia 2004; Dodd *et al.* 2008; Chen *et al.* 2011; Zou *et al.* 2014) without changing *γ*_j_ in Cx50 GJ (Chen *et al.* 2011). Another possibility is that intracellular Ca^2+^ could directly bind to the GJ pore lining residues to block/reduce ion permeation. This idea was supported by a structural study with a total of 12 Ca^2+^-binding sites mapped onto residues (E42, G45, and E47) at the border of M1–E1 domains in the Cx26 subunit (Bennett *et al.* 2016). Binding one or more Ca^2+^ in a GJ could drastically change the pore electrostatic properties (Bennett *et al.* 2016). Another study showed evidence that Ca^2+^ binds to D50 and E47 of Cx26 to gate un-apposed hemichannel to a closed state (Lopez *et al.* 2016). The region of the M1 and E1 border is highly conserved with a cluster of negatively charged residues, which could serve to bind divalent cations including Ca^2+^ and possibly Mg^2+^. Supporting this divalent cation binding model, the [Mg^2+^]_i_-dependent increase in coupling conductance of Cx36 GJ was also mapped to a residue in this M1–E1 region (Palacios-Prado *et al.* 2014). In addition, Cx37 and Cx50 GJ channels showed a decrease in *γ*_j_ with elevation of [Mg^2+^]_i_ (Banach *et al.* 2000; Tejada *et al.* 2018). The results from our current study are consistent with a possible binding of Mg^2+^ to this region of Cx46 and Cx50 GJ to reduce differentially the rate of ion permeation (or *γ*_j_). Molecular dynamic studies showed that divalent cations showed an increased dwell time at residues (E47 and D50) at the M1–E1 region in Cx26 (Zonta *et al.* 2014; Lopez *et al.* 2016). Structural and molecular dynamic studies in the presence of Mg^2+^ would help to further reveal the mechanisms of how Mg^2+^ modulates these lens GJs.

The single channel conductance (*γ*_j_) differences of Cx46/Cx50 GJ channels in our study in the presence of Mg^2+^ are different from those reported previously (Hopperstad *et al.* 2000). We do not have a simple explanation, but possibilities could include inter-species differences between sheep connexins (from current study) vs. the rodent connexins (used in the previous study), differences in patch pipette solution, and/or differences in conventions and patch clamp handling procedures *etc*. This should be verified in future studies, ideally from the same laboratory.

Cx46, Cx50, and most of our designed Cx46 and Cx50 variants expressed well in GJ-deficient N2A cells and were able to form functional GJs as both single channel and macroscopic GJ currents were recorded. To optimize the yield of recording cell pairs with only 1–2 functional GJs, we lowered the replating time to as low as 30 minutes on the recording day. Even with this optimized condition, the yield of getting GJ single channel current was still very low (less than 10% of total recorded cell pairs). With this low yield, we could not test systematically for different doses of [Mg^2+^]_i_ to construct dose–inhibition curves for proper pharmacological analysis. The Cx50 F43E did show single channel currents but rarely stayed at the fully open state in the Mg^2+^-free patch pipette solution. In the presence of 5 mmol/L [Mg^2+^]_i_, we could not record any functional GJs. Most likely the Cx50 F43E GJs stayed in a fully closed state and were unable to transition to a fully open state.

During the data collection of single heterotypic Cx46/Cx50 GJ currents, we also recorded many cell pairs with macroscopic GJ currents in response to different *V*_j_s. However, we noticed that the peak amplitude of macroscopic GJ currents was not stable and could not be used as a good representation of the single channel currents at the main conductance state, specifically the rectification property was not clear in these macroscopic GJ currents at different *V*_j_s. The main reason for this apparent discrepancy was that the peak macroscopic currents at the relatively high *V*_j_s (±60 to ±100 mV) were not very stable due to incomplete recovery from the previous *V*_j_-induced *V*_j_-gating (which pushed a lot of functional GJs into residue conductance or fully closed states and likely only a portion of these gated channels was recovered at the given inter pulse interval) as well as the differences in gating kinetics of GJ deactivation during the *V*_j_ pulse. We tried to increase the inter pulse interval to allow a longer time for improved recovery from deactivated GJ channels but failed to resolve the macroscopic current stability issue as sometimes the GJ currents ‘run down’ and/or are not stable during the prolonged period (*e*.*g*., change in access for the whole recording and/or leak changes at the patch *etc*.). This is the reason we only focused on quantitative analysis of single channel GJ currents at the main conductance state in this study, which is a much more reliable parameter for heterotypic GJ channels.

In conclusion, we showed that the elevation of intracellular Mg^2+^ to 5 mmol/L led to a differential reduction in the *γ*_j_s of Cx46 and Cx50 GJs. The relatively higher reduction in Cx46 *γ*_j_ than in Cx50 *γ*_j_ by Mg^2+^ caused rectification in the *γ*_j_ of heterotypic Cx46/Cx50 GJ.

## METHOD

### Plasmid construction

We used expression constructs (pIRES2) with an untagged fluorescent reporter gene, encoding either EGFP (pIRES2-EGFP) or DsRed (pIRES2-DsRed2). Sheep Cx50-IRES-GFP (also known as Cx49 or sCx50), sheep Cx46-IRES-GFP (also known as Cx44) and Cx46-IRES-DsRed were generated as previously described (Yue *et al.* 2021; Jaradat *et al.* 2022). Briefly, the cDNAs of Cx50 or Cx46 were synthesized and each of them was inserted into the expression vector at the restriction enzyme sites, XhoI and EcoRI (NorClone Biotech Laboratories, London, Ontario). These vectors were used as templates to generate point mutations, Cx46 E43F and Cx50 F43E, with the following primers:

Cx46 E43F: forward 5’ GCC GCG GCC GAG TTC GTG TGG GGG GAT GAG 3’ and reverse 5’ CTC ATC CCC CCA CAC GAA CTC GGC CGC GGC 3’

Cx50 F43E: forward 5’ GGT ACT GCT GCA GAC GAG GTG TGG GGG GAT GAG 3’ and reverse 5’ CTC ATC CCC CCA CAC CTC GTC TGC AGC AGT ACC 3’

### Cell culture and transient transfection

Gap junction deficient neuroblastoma (N2A) cells (American Type Culture Collection, Manassas, VA) were cultured and grown in Dulbecco’s modified Eagle’s medium (DMEM) containing 4.5 g/L D-(+)-glucose, 584 mg/L L-glutamine (4 mmol/L), 110 mg/L sodium pyruvate, 10% fetal bovine serum (FBS), 1% penicillin (100 units/mL), and 1% streptomycin (100 μg/mL or 172 μmol/L), in an incubator with 5% CO_2_ at 37 °C (Sun *et al.* 2013). N2A cells at ~60% confluence were transfected with 1.0 μg of a cDNA construct and 2 μL of X-tremeGENE HP DNA transfection reagent (Roche Diagnostics GmbH, Indianapolis, IN, USA) in a regular medium overnight. Next, morning the transfected cells were replated onto coverslips for 0.5–1.5 h prior to recording. For heterotypic GJs, cells transfected with Cx46-IRES-DsRed were mixed with cells transfected with Cx50-IRES-EGFP before replating onto coverslips for 0.5–1.5 h. Cell pairs with one red and the other green were selected for dual patch clamp recording.

### Electrophysiological recording

Glass coverslips with transfected cells were placed into a recording chamber one at a time on an inverted fluorescent microscope (DMIRB, Leica). The chamber was filled with extracellular solution (ECS) at room temperature (21–24 °C). The ECS contained (in mmol/L): 135 NaCl, 2 CsCl, 2 CaCl_2_, 1 MgCl_2_, 1 BaCl_2_, 10 HEPES, 5 KCl, 5 D-(+)-glucose, 2 Sodium pyruvate, pH 7.4 (adjusted with 1 mol/L NaOH), and with an osmolarity of 310–320 mOsm. Patch pipettes were pulled using a micropipette puller (PC-100, Narishige International USA Inc., Amityville, NY, USA) and filled with intracellular solution (ICS). The Mg^2+^-free ICS contains (in mmol/L): 130 CsCl, 10 EGTA, 0.5 CaCl_2_, 5 Na_2_ATP, and 10 HEPES, adjusted to pH 7.2 with 1 mol/L CsOH, and with an osmolarity of 290–300 mOsm. For Mg^2+^ containing ICS, 5 mmol/L MgATP was used to replace Na_2_ATP. Isolated heterotypic red/green cell pairs (GFP and DsRed reporter expressing cell pairs) were selected to study heterotypic Cx46/Cx50 GJs. For homotypic GJ studies, isolated GFP expressing cell pairs were selected.

### Single channel recording and analysis

After whole cell recording was established, both cells were initially voltage clamped at 0 mV with Axopatch 200B amplifiers (Molecular Devices, Sunnyvale, CA, USA). A series of voltage pulses (±20 to ±100 mV with 20 mV increment and 7 s duration) were administered to one of the cells to establish transjunctional voltage (*V*_j_). The other cell was held at 0 mV to record unitary transjunctional current (*i*_j_). The *i*_j_ was low-pass filtered (cut-off frequency at 1 kHz) at the amplifier, digitized via an AD/DA converter at a sampling rate of 10 kHz (Digidata 1322A, Molecular Devices, Sunnyvale, CA, USA), and recorded with Clampex9.2 software (pClamp9.2, Molecular Devices, Sunnyvale, CA, USA).

Single gap junctional currents (*i*_j_s) could be observed in cell pairs with few active channels. The *i*_j_s were further filtered using a low-pass Gaussian filter at 200 Hz in Clampfit 9.2 for measuring current amplitude and display in figures. The amplitude *i*_j_ values for the fully open state at different *V*_j_s were measured by either fitting Gaussian functions on all-point current amplitude histograms or directly reading manually in Clampfit. For homotypic Cx46, Cx50, and their variant GJs, the *i*_j_ values of the same cell pair with two *V*_j_ polarity were averaged and then plotted with each tested *V*_j_s to generate *i*_j_–*V*_j_ plot for each cell pair. Linear regression of the *i*_j_–*V*_j_ plot for each cell pair was used to estimate slope unitary GJ channel conductance (also known as slope single channel conductance, *γ*_j_). The slope *γ*_j_s of different cell pairs were averaged and presented as bar graphs. For heterotypic Cx46 / Cx50 GJs, two *V*_j_ polarities were individually defined and analyzed. When the Cx46 expressing cell with +*V*_j_s (or the Cx50 expressing cell with –*V*_j_s), the *V*_j_ polarity was defined as Cx46+. Reversely, when the Cx46 expressing cell with –*V*_j_s (or the Cx50 expressing cell with +*V*_j_s), the *V*_j_ polarity was defined as Cx46–. The *i*_j_s of both Cx46+ and Cx46– polarities were analyzed separately and *i*_j_–*V*_j_ plots were generated to obtain slope *γ*_j_s for each of these polarities. Slope *γ*_j_s were averaged and plotted as bar graphs for statistical comparison.

### Statistical analysis

Data in *i*_j_–*V*_j_ plots and bar graphs represent mean ± SEM. Paired Student *t*-test was used to compare slope *γ*_j_s of Cx46+ and Cx46– *V*_j_ polarity in heterotypic Cx46/Cx50 GJs. The unpaired *t*-test was used to compare *γ*_j_ values of homotypic GJs of different connexins, their variants, and/or under different [Mg^2+^]_i_ concentrations. Statistical significance is indicated with the asterisks on the graphs for group comparisons (*P < 0.05; **P < 0.01; or ***P < 0.001).

## Conflict of interest

Honghong Chen and Donglin Bai declare that they have no conflict of interest.
